# The impact of armed conflicts on HIV treatment outcomes in Sub-Saharan Africa: a systematic review and meta-analysis

**DOI:** 10.1186/s13031-024-00591-8

**Published:** 2024-05-17

**Authors:** Hafte Kahsay Kebede, Hailay Abrha Gesesew, Amanuel Tesfay Gebremedhin, Paul Ward

**Affiliations:** 1 Tigray Health Research Institute, Mekelle, Tigray, Ethiopia; 2 College of Health Sciences, Mekelle University, Mekelle, 231 Ethiopia; 3 Research Centre for Public Health, Equity, and Human Flourishing, Torrens University Australia, Adelaide, 5000 Australia; 4 Curtin School of Population Health, Curtin University, Bentley, WA Australia; 5 School of Nursing and Midwifery, Edith Cowan University, Perth, Australia

**Keywords:** AIDS, Armed conflict, HIV, Sub-Saharan Africa

## Abstract

**Background:**

Despite the fact that Sub-Saharan Africa bears a disproportionate burden of armed conflicts and HIV infection, there has been inadequate synthesis of the impact of armed conflict on HIV treatment outcomes. We summarized the available evidence on the impact of armed conflicts on HIV treatment outcomes in Sub-Saharan Africa from 2002 to 2022.

**Methods:**

We searched four databases; MEDLINE, PubMed, CINHAL, and Scopus. We also explored grey literature sources and reviewed the bibliographies of all articles to identify any additional relevant studies. We included quantitative studies published in English from January 1, 2002 to December 30, 2022 that reported on HIV treatment outcomes for patients receiving antiretroviral therapy (ART) in conflict and post-conflict areas, IDP centers, or refugee camps, and reported on their treatment outcomes from sub-Saharan Africa. Studies published in languages other than English, reporting on non-ART patients and reporting on current or former military populations were excluded. We used EndNote X9 and Covidence to remove duplicates, extracted data using JBI-MAStARI, assessed risk of bias using AHRQ criteria, reported results using PRISMA checklist, and determined Statistical heterogeneity using Cochran Q test and Higgins I^2^, R- and RevMan-5 software were used for meta-analysis.

**Results:**

The review included 16 studies with participant numbers ranging from 102 to 2572. Lost To Follow-Up (LTFU) percentages varied between 5.4% and 43.5%, virologic non-suppression rates ranged from 25 to 33%, adherence rates were over 88%, and mortality rates were between 4.2% and 13%. A pooled meta-analysis of virologic non-suppression rates from active conflict settings revealed a non-suppression rate of 30% (0.30 (0.26–0.33), I2 = 0.00%, *p* = 0.000). In contrast, a pooled meta-analysis of predictors of loss to follow-up (LTFU) from post-conflict settings identified a higher odds ratio for females compared to males (1.51 (1.05, 2.17), I2 = 0%, *p* = 0.03).

**Conclusion:**

The review highlights a lack of research on the relationship between armed conflicts and HIV care outcomes in SSA. The available documents lack quality of designs and data sources, and the depth and diversity of subjects covered.

**Supplementary Information:**

The online version contains supplementary material available at 10.1186/s13031-024-00591-8.

## Introduction

Armed conflicts have a negative impact on the health of populations, particularly on HIV patients who require strict adherence to treatment [1–7]. Providing regular HIV care and ensuring a continuous supply of drugs in conflict zones is difficult [2], leading to increased HIV incidence, prevalence, and related morbidity and mortality[8,9]. Less than 20% of people in conflict situations receive ART [10], and HIV services are scarce or nonexistent in conflict-affected countries; such as the Central African Republic (CAR), South Sudan, and parts of Yemen [10]. With 1.8 billion people living in conflict-affected areas [11], and 89.3 million people displaced from their homes by the end of 2021[12], researchers need to investigate how war affects HIV treatment.

However, there is a lack of systematic reviews investigating the connection between HIV and conflict, including the impact of armed conflict on HIV treatment outcomes. There were few systematic reviews[9,13–17] that have paid close attention to HIV prevalence and incidence as a result of armed conflict. None of the systematic reviews assessed the impact of armed conflict on HIV treatment outcomes. Given the importance of HIV treatment in reducing HIV incidence and prevalence, the double burden of HIV and conflict in Sub-Saharan Africa, and the already fragile healthcare system in the region, there is a need for systematic synthesis and exploration of this area of research. This review aims to synthesize the impact of armed conflict on HIV treatment outcomes among HIV patients affected by armed conflicts in SSA, including retention, attrition, LTFU, clinical failure, immunological failure, treatment failure, virological failure, and mortality.

## Methods

### Study registration

The review was registered in the International Prospective Register for Systematic Reviews (PROSPERO), with the registration number CRD42022361924 and the protocol is published online in BMJ Open [18].

### Population and context

**Fig. 1:**
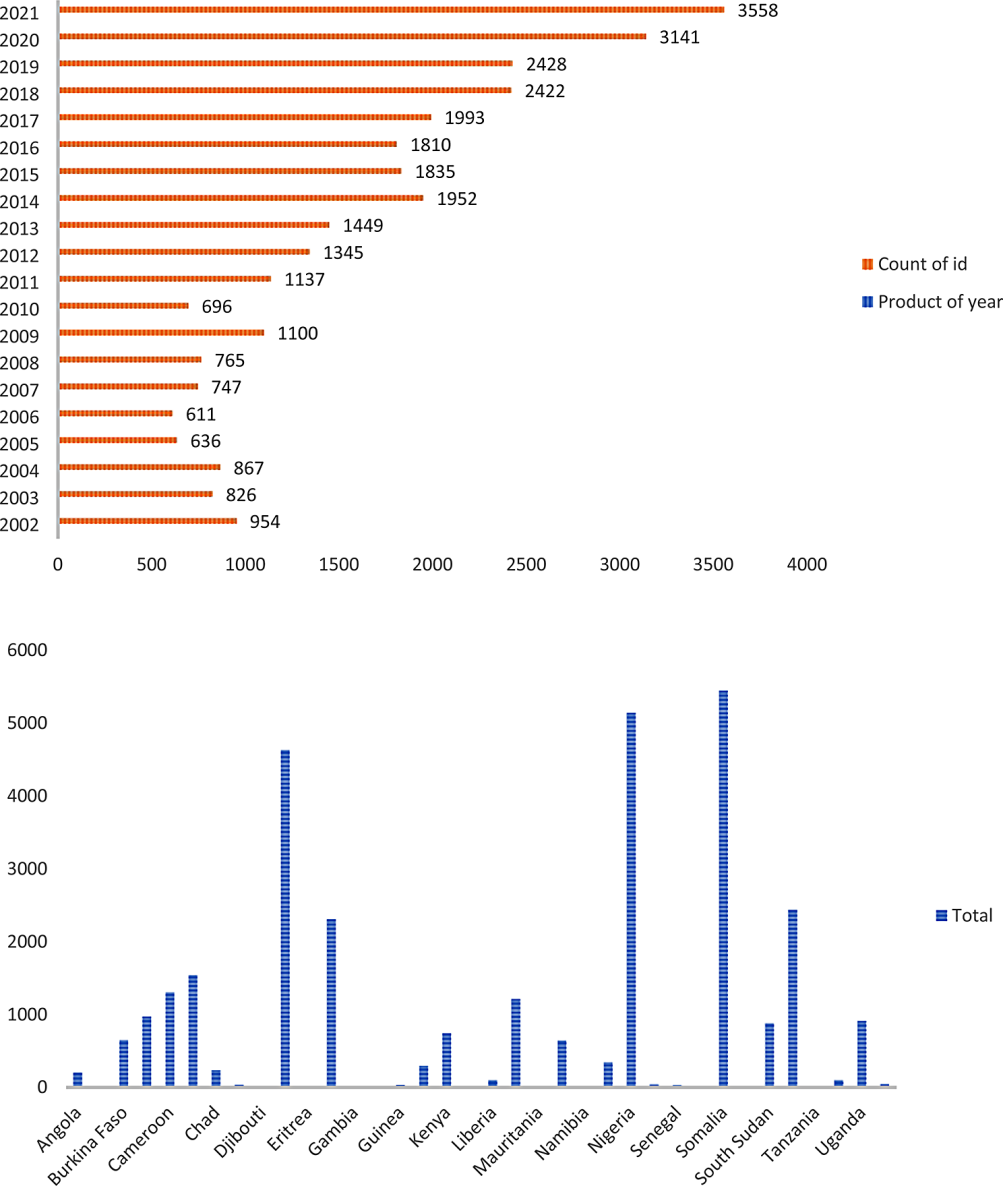
Number of conflict-affected and conflict incidents in sub-Saharan Africa between 2002 and 2021. (Source: UCDP Version 2021)

The review included individuals in sub-Saharan Africa who received antiretroviral therapy between 2002 and 2022 and were living in conflict-affected, post-conflict, or displacement areas. We selected 2002, as many sub-Saharan African countries introduced HIV care and treatment programs in 2002[19]. The review was limited to sub-Saharan Africa due to the region’s disproportionately high affected by both HIV infection [20] and armed conflicts[16,21–24] (Fig. 1), weak healthcare systems, and lack of clear directives and funding for addressing HIV care in conflict-affected settings.

### Search strategy and data sources

The search was conducted using three themes: conflict, HIV care, and SSA, and was conducted in four databases. Although the initial plan was to search five databases, access to Web of Sciences was not possible. After seeking advice from our librarian, we chose to exclude Web of Sciences from our search since it had comparable literature content to SCOPUS. Consequently, we searched the remaining four databases, which included MEDLINE, PubMed, CINHAL, and SCOPUS. We also explored grey literature sources and reviewed the bibliographies of all articles to identify any additional relevant studies.

### Study selection and eligibility criteria

The study only included quantitative studies conducted in English from January 1, 2002 and December 31, 2022 that focused on HIV patients receiving ART from conflict areas, IDP centers, or refugee camps, and reported on their treatment outcomes. Studies that included both conflict-affected and non-conflict-affected populations were only included if they provided data specifically for the conflict-affected group. Studies published in languages other than English, those that did not mention conflict or provide disaggregated data for conflict-affected populations, studies that involved current or former military populations, studies from countries outside of SSA, studies reporting on African refugees settled outside of SSA, studies published before 2002, and studies reporting on HIV patients not receiving ART were excluded.

### Quality assessment

The search results were filtered through a multi-step process that included independent review by two reviewers, evaluation of methodological validity using standardized JBI appraisal instruments [25], and assessment of risk of bias using AHRQ criteria [26]. Disagreements among reviewers were resolved through discussion and consensus but included and extracted data from all relevant documents irrespective of their quality score.

### Data extraction

We used EndNote X9 and Covidence to remove duplicates. Data were extracted using the standardized data extraction tool from JBI-MAStARI [27]. The data extracted included specific details about the authors, publication year, country of study, populations, sample size, summary of HIV treatment outcome.

### Types of interventions and comparators

The review considered impact of armed conflict on HIV treatment outcomes.

### Types of outcome measures

The study focused on HIV care treatment outcomes, including adherence, LTFU, clinical failure, immunological failure, virological failure, and mortality.

### Operational definitions

There is no universally accepted definition of armed conflict, as it is an umbrella word for a wide range of ideas and activities, such as conflict, war, violence, terrorism, or catastrophic loss of civilian life, a civil unrest, massive displacement, and violations of human rights and international humanitarian law. While we put the definition of conflict as follows, we would like to remain open to additional definitions by authors.

#### Armed conflict

Is a situation in which states or other organized parties fight against each other by way of military force. Armed conflicts shall be of international and non-international armed conflicts [28].

#### Conflict *affected areas*

Are areas experiencing an armed conflict, and post-conflict regions. The area may be a region, a country, an area within a country, or an area that crosses one or more country boundaries. The impact of conflict may extend beyond the region of conflict to include surrounding areas and those hosting displaced persons. As a result, our definition remains open to the definition of authors.

#### Conflict *-affected populations*

Individuals, groups, and communities affected by and remaining in conflict-affected areas, as well as those forcibly displaced from them as refugees and IDPs. Conflict may have an impact on those who host IDPs and refugees. As a result, our definition remains open to author definition.

#### Internally displaced persons (IDPs)

Those who have been forced or compelled to leave their homes, often due to armed conflict, and who stay within their country’s borders.

#### Post-conflict

Refers to the period immediately following a conflict, when open combat is over. Nevertheless, despite its linguistic simplicity, the phrase is more challenging to define practically in terms of time, response, transformation, and sustainability. Therefore, our definition remains open to author definition.

#### Refugees

People who are outside their country of birth due to conflict, war, widespread violence, or feared persecution and who as a result need international protection within the African continent [29].

### Data management, analysis and synthesis

The results were reported in accordance with the Preferred Reporting Items for Systematic Reviews and Meta-Analyses statement [30] and meta regression was done to assess the relationship between armed conflict and HIV treatment outcomes. The Cochran Q test and Higgins I^2^[31] were used to determine statistical heterogeneity, and the random effect model was used when moderate statistical heterogeneity was detected. A meta-analysis to assess the predictors of LTFU was also conducted. The study used various statistical tools and software for analysis; R- software, STATA, and RevMan-5.

## Result

### Outcome of the search

**Fig. 2:**
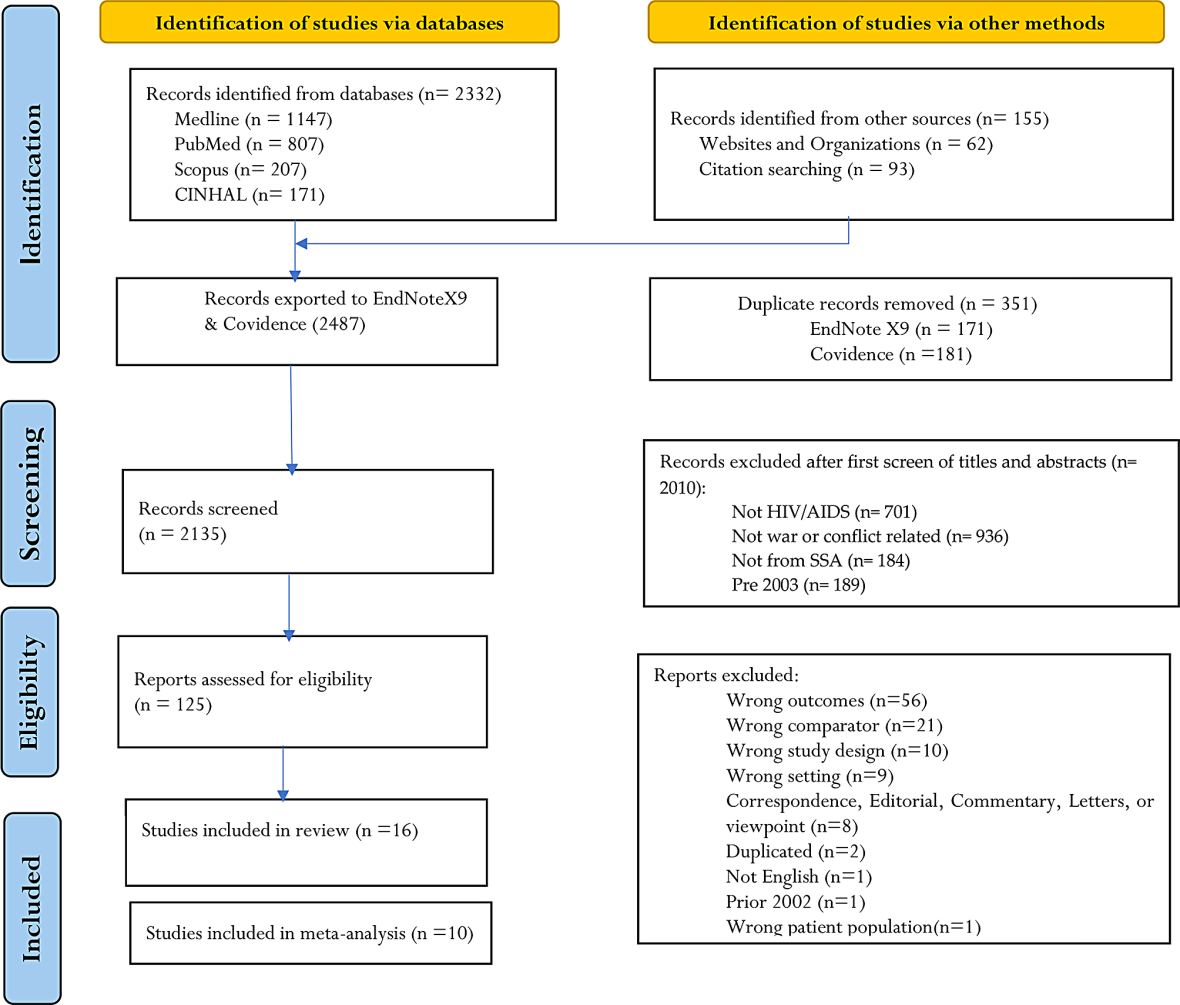
PRISMA flowchart diagram of study selection impact of armed conflict on HIV care outcomes in SSA, 2002–2022

The comprehensive search identified 2487 records; 2332 identified through database searches, and 155 through manual searching of references and websites identified. All the identified 2487 references were exported into EndNote X9, and then into Covidence; 171 in the endnote, and 181 articles in the Covidence duplications were removed. After eliminating duplicates and conducting a comprehensive screening using Covidence was done, and 16 studies were considered eligible for data extraction, with 10 of them providing data for meta-analysis (Fig. 2).

### Description of included studies

The research covered in the review spanned from 2007[32] to 2022[33] and had varying follow-up periods, ranging from 4 days [34] to 60 months [35]. The majority of the studies were conducted in Western and Eastern Africa, with a significant number of studies coming from the Democratic Republic of the Congo[32,33,35–37], Kenya [38–40], and Uganda[34,41,42]. Most of the studies were designed as cohort studies, either retrospective[32,35,38–41,43–45] or prospective[33,36,37,42,46]. The majority of the studies focused on adult populations[33–37,39,40,42–44], with one study [38] specifically examining children. The studies included patients who were non-IDPs[32,33,35–38,40,41], non-IDPs & IDPs[34,39,42,44,45], refugees[44,47], or a mix of these groups [43]. Ten studies were carried out in conflict settings[32–34,36,37,42,44–47], while five took place in post-conflict zones[35,38–41], and one in both [43] (Table 1 and Supplementary Table 1).

**Table 1:** Summary of characteristics of included studies from SSA, 2002–2022

Characteristics	References	Number (n)
***Study design***		
Cohort	[32,33,35–46]	14
Retrospective	[32,35,38–41,43–45]	9
Prospective	[33,36,37,42,46]	5
Case study	[47]	1
Cross sectional	[34]	1
***Geographic region***		
West Africa		
CAR	[46,47]	2
DRC	[32,33,35–37]	5
East Africa		
Kenya	[38–40]	3
Uganda	[34,41,42]	3
South Sudan	[44]	1
Multiple regions	[43,45]	2
***Context***		
-Active conflict setting	[32–34,36,37,42,44–47]	9
-Post conflict setting	[35,38–41]	5
-Both	[43]	1
***Treatment outcomes***		
Adherence	[32,34,38,42,44]	5
-Only adherence	[34,44]	2
Treatment interruption	[39,40]	2
-only TI	-	0
LTFU	[32,35,36,38,41,43,45–47]	9
-Only LTFU	[35,36]	2
Immunologic gain	[32,40,42,43]	4
-only IG	-	0
Viral load	[37,40,45,47]	4
-only VL	[37]	1
Drug resistance	[40]	1
Mortality	[32,33,42,43,46,47]	6
-only mortality	[33]	1
***Participants/ target population***		
Adults	[33–37,39,40,42–44],	10
General population	[32,41,45–47]	5
Children/pediatrics	[38]	1
PMCTC	-	0
***Status of participants***		
Non-IDPs	[32,33,35–38,40,41]	8
IDPs and non-IDPs	[34,39,42,44,45]	5
Refuges	[44,47]	2
IDPs, non-IDPs, and refugees	[43]	1
***NGO funded***		
Yes	[32,34,35,38–47]	13
No	[33,36,37]	3

### Quality assessment—Data aource/Validity/Reliability

Methodological validity of the included studies was evaluated using JBI appraisal instruments. The quality of evidence was rated as high quality (*n* = 7, 90.91%, *n* = 5, 81.82%, *n* = 1, 100%, *n* = 1, 90%, *n* = 1, 75%, *n* = 1, 72.73%) and presented moderate quality. The outcomes of the BJI assessment are shown in supplementary (supplementary Table 2).

### Conflict and HIV treatment outcomes

The studies reported various outcomes, with four[33,36,37,46] focusing on the relationship between conflict and HIV treatment outcome as the primary outcome, five reporting adherence rates[32,34,38,42,44], two reporting treatment interruption[39,40], nine reporting LTFU[32,35,36,38,41,43,45–47], four reporting immunologic gain[32,40,42,43], four looking at the rate of viral non-suppression[37,40,45,47], and seven reporting mortality[32,33,35,42,43,46,47] (Supplementary Table 3).

### Adherence

The review found that adherence rates among HIV patients in Sub-Saharan Africa ranged from 88.2%[44] to 99.6%[34]. The studies reported predictors of non-adherence, and non-adherence was associated with being on first-line therapy(OR = 22.22, 95% CI 1.53, 333.33; *p* = 0.02)[34], and feeling condemned by clinic workers (OR = 22.22, 95%CI 1.53, 333.33; *p* = 0.02)[34].

#### Pooled meta-analysis

**Fig. 3:**
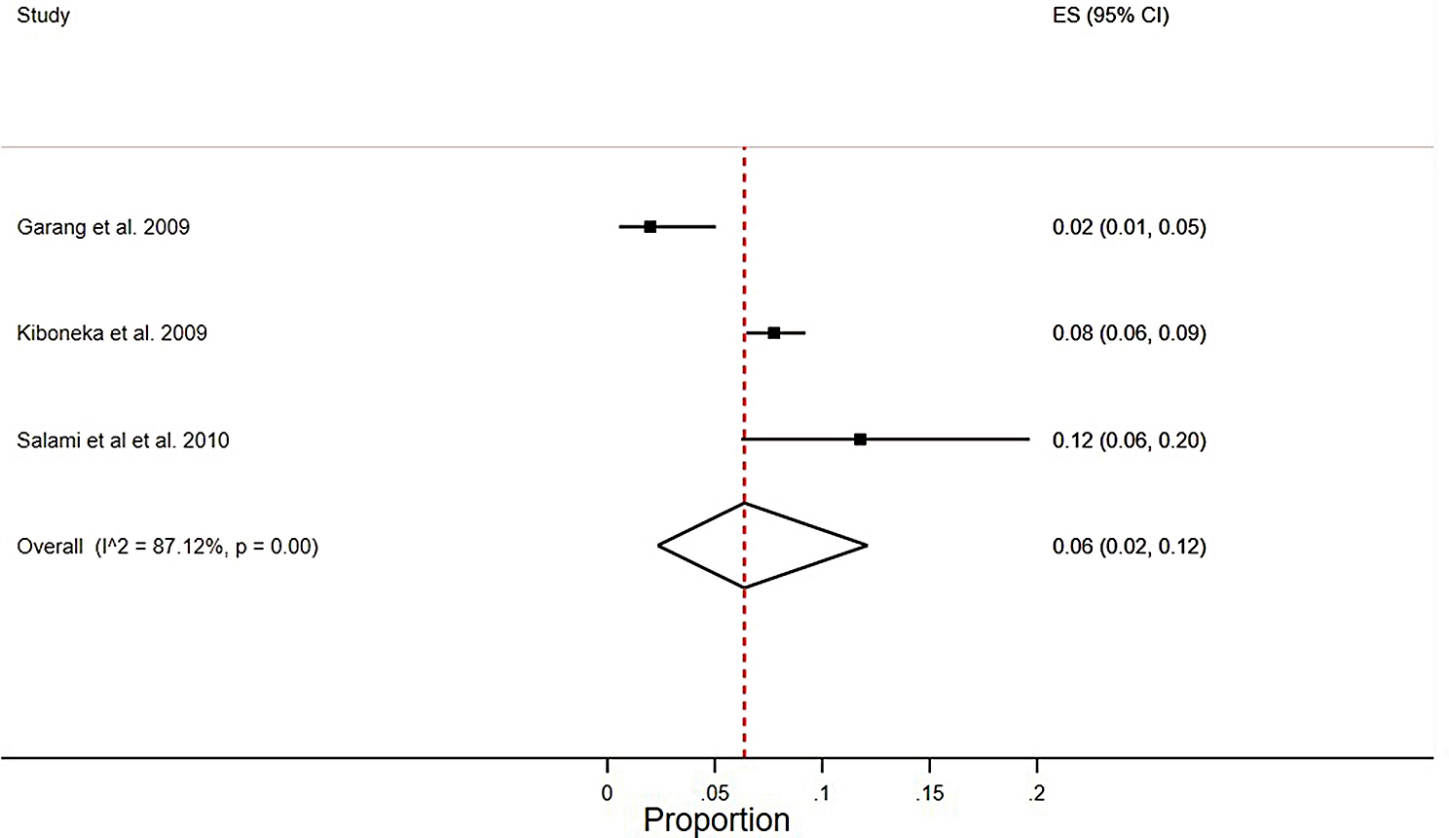
Pooled meta-analysis of non-adherence rate from active conflict settings among HIV patients who were on ART in SSA from 2002 to 2022

The pooled adherence rate among HIV patients in conflict-affected SSA regions was 0.06 (0.02–0.12), I2 = 87.12%, *p* = 0.00, with a significant clinical heterogeneity (Fig. 3).

### Treatment interruption (TI)

Two studies[39,40] conducted in Kenya reported instances of treatment interruption, with the risk of interruption increasing by 71% during post-election violence (95%CI 34, 118, *p* = 0.001). Male patients (OR = 1.37, 95%CI 1.07, 1.76; *p* = 0.01), and who traveled more than three hours to the clinic (OR = 1.86, 95%CI 1.28, 2.71; *p* = 0.001) were found to be at a higher risk of treatment interruption [39]. Treatment interruptions were associated with detectable viral load, with viral loads exceeding 5,000 copies/mL and 10,00060 copies/mL[40].

### Lost to follow-up

In active conflict settings, the rate of loss to follow-up (LTFU) ranged from 5.4% (95% CI = 3.2–7.5)[32] to 28.8% (95% CI: 24.9–33.1) [36], while post-conflict areas had a paradoxical level of LTFU ranging from 2.6%[38] to 43.5%[41]. The included studies indicated that factors like educational level [36], place of residence [36], prior experience with antiretroviral therapy (ART) upon enrollment [36], and the World Health Organization (WHO) clinical stage of disease [41] were linked to loss to follow-up (LTFU) among adult patients. Additionally, the study showed that receiving ART reduced the likelihood of complete loss to follow-up among children with HIV in post-crisis Kenya [38].

#### Pooled meta-analysis

**Fig. 4:**
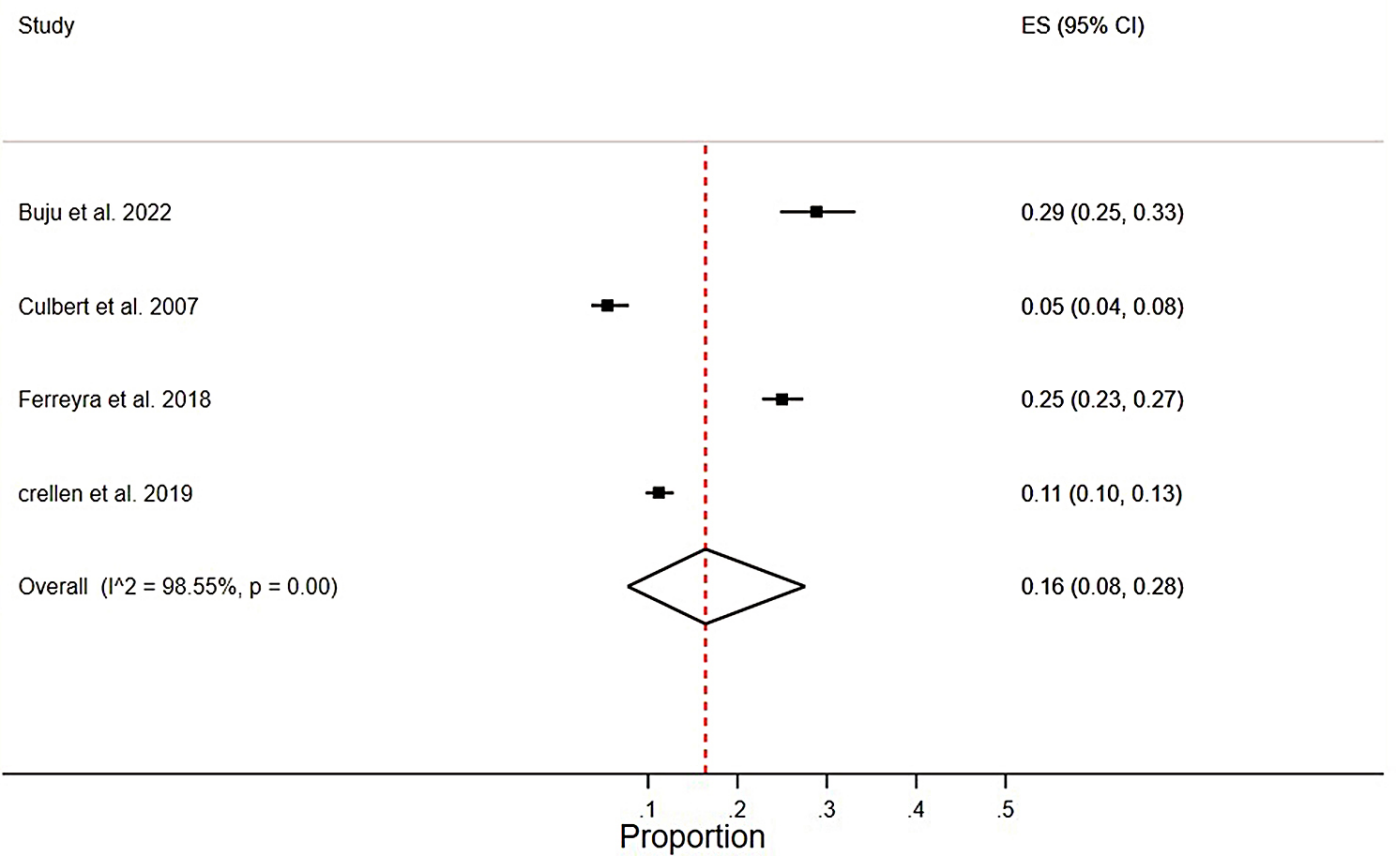
Pooled meta-analysis of LTFU rate from active conflict settings among HIV patients who were on ART in SSA from 2002 to 2022

**Fig. 5:**
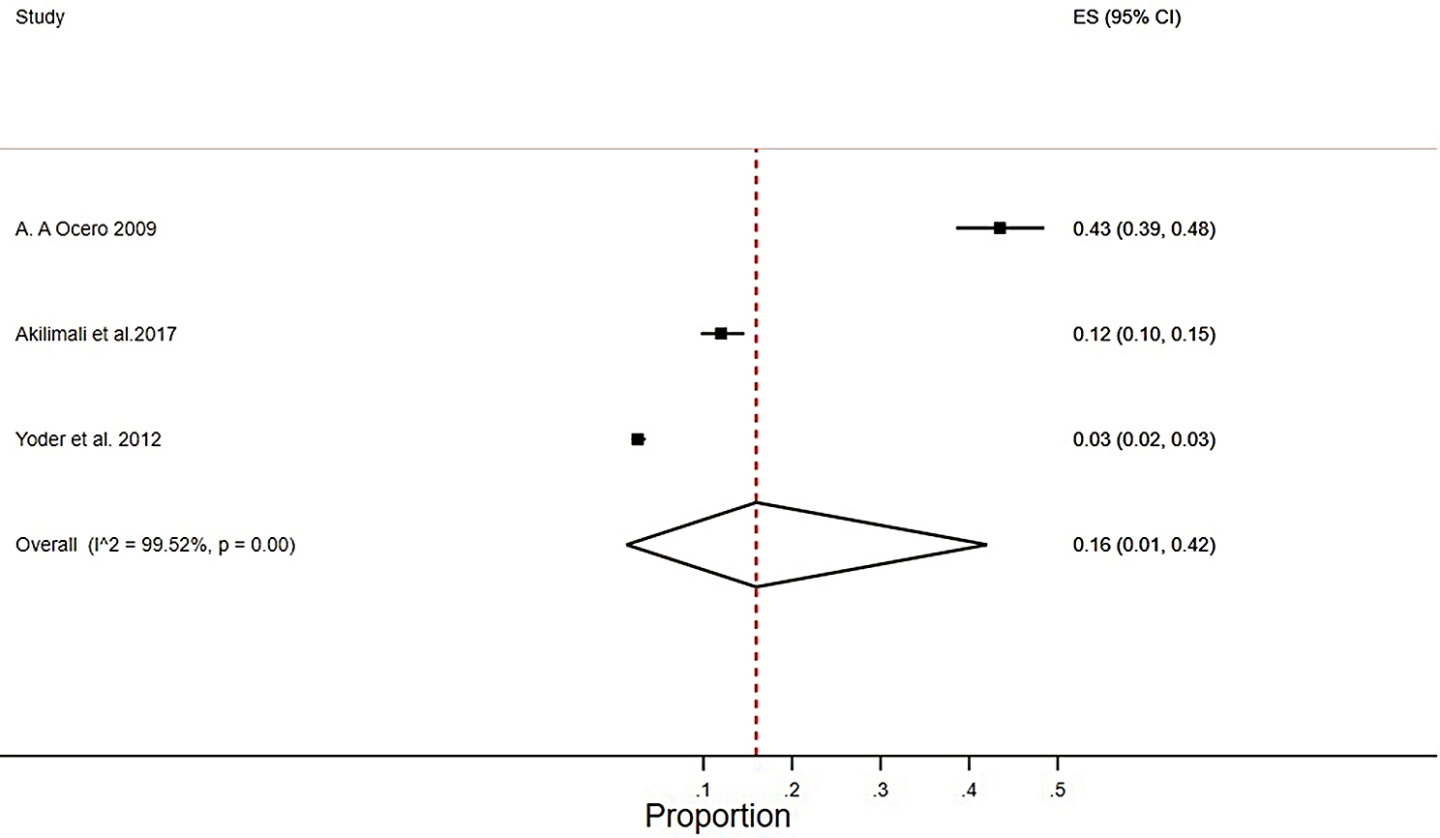
Pooled meta-analysis of LTFU rate from post conflict settings among HIV patients who were on ART in SSA from 2002 to 2022

Pooled LTFU rates of 0.16 (0.08–0.28), I2 = 98.55%, *p* = 0.00 were found in studies from active conflict settings[32,36,46,47], (Fig. 4), and 0.16(0.01–0.42), I2 = 99.52%, *p* = 0.00 post-conflict settings[35,38,41] (Fig. 5).

**Fig. 6:**
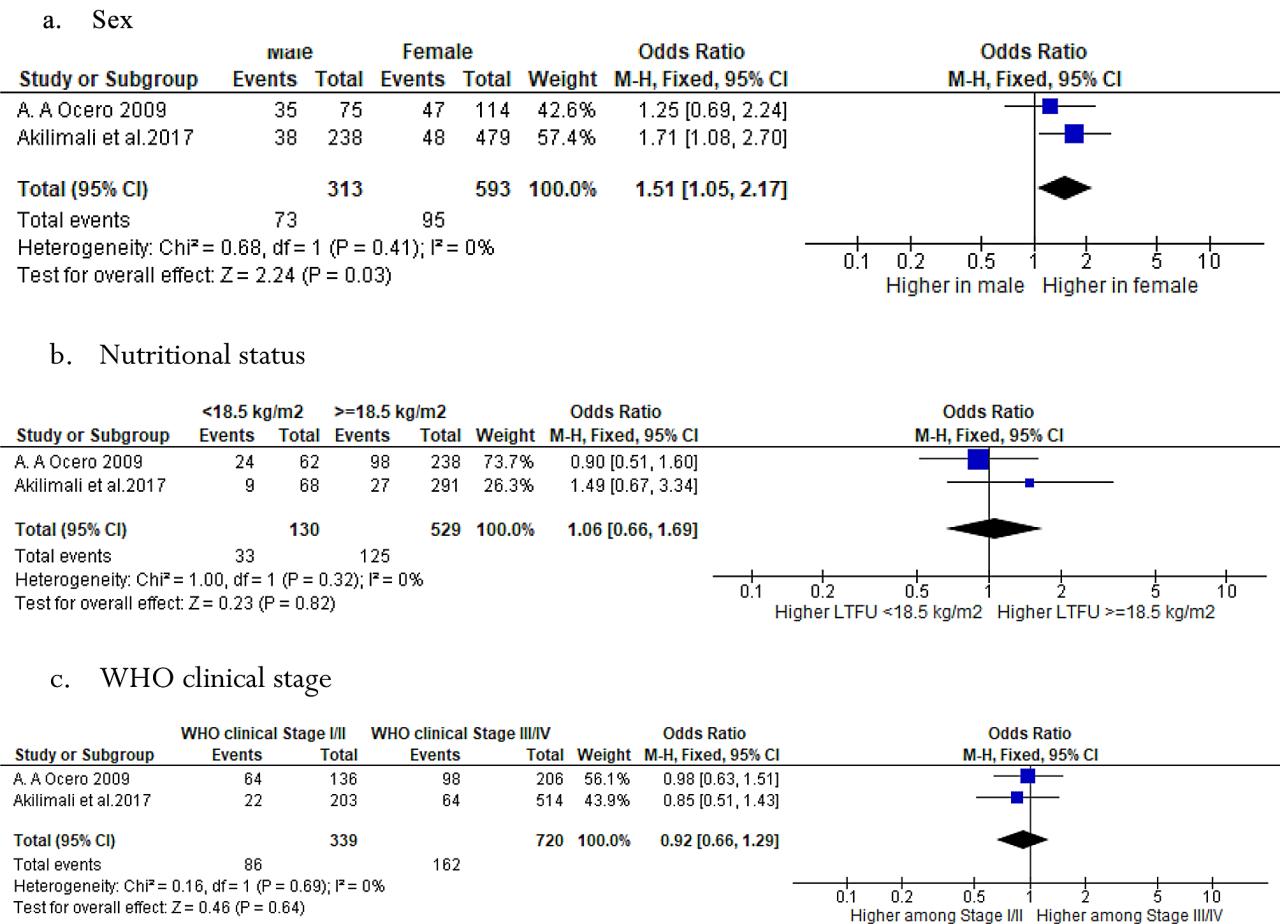
Meta-analysis of predictors of LTFU in post conflict-settings among HIV patients who were on ART in SSA from 2002 to 2022

Similarly, a review of studies conducted in post-conflict settings analyzed factors that may predict loss to follow-up (LTFU) in HIV care. The study found that only gender was statistically significant, with a 1.51 (1.05, 2.17), I^2^ = 0%, *P* = 0.03 odds ratio for LTFU in females compared to males (Fig. 6).

### Immunologic gain

CD4 gain was reported by four studies[32,40,42,43], as a secondary outcome, with changes ranging from 129 mm3[43] to a median of 163 cells/mm^3^[32] in six months.

### Viral non-suppressions

Four studies examined the prevalence of viral non-suppression[37,40,45,47], with different thresholds ranging from > 50[45,47] to > 1000 copies/mL[45,47]. Three studies[37,45,47] investigated predictors of viral non-suppression, with two finding no significant variables and one [37] reporting that being in stage III or IV of the disease (AOR = 1.86, 95% CI 1.01–3.43), and having a high baseline HIV viremia of over 1000 copies/mL(AOR = 3.41, 95% CI 1.64–7.08) were associated with increased risk [37].

#### Pooled meta-analysis

**Fig. 7:**
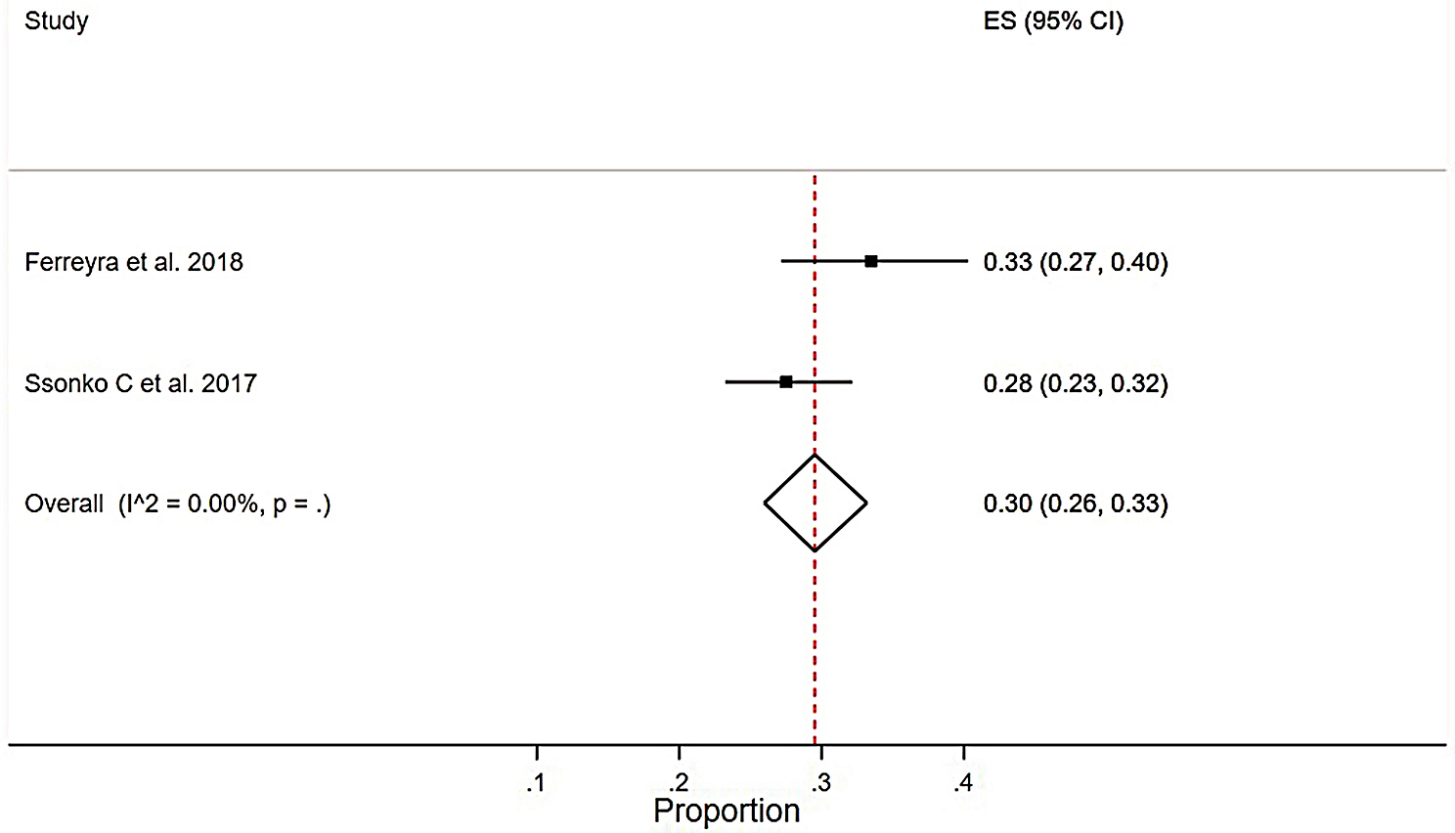
Pooled meta-analysis of virologic non-suppression rate from active conflict settings among HIV patients who were on ART in SSA from 2002 to 2022

A pooled-analysis of two studies[45,47] from active conflict settings found a pooled non-suppression rate of 30% (0.30(0.26–0.33), I2 = 0.00%, *p* = 0.000) using a cut-off point of > 1000 copies/mL and a follow-up period of 6–12 months (Fig. 7).

### Mortality

Seven studies provided information on mortality rates, with five conducted in active conflict settings[32,33,42,46,47], and one each in post-conflict [35] and both conflict and post-conflict [43] settings. Mortality rates ranged from 3.6% (17/468)[33] to 13.0% (182/1400)[47]. Three studies[33,46,42] examined predictors of mortality namely, socio-demographic variables such as age and sex, as well as clinical variables such as baseline ART status, viral load, CD4 count, WHO clinical stage, and adherence level were reported having associated with lower risk of mortality.

#### Pooled meta-analysis

**Fig. 8:**
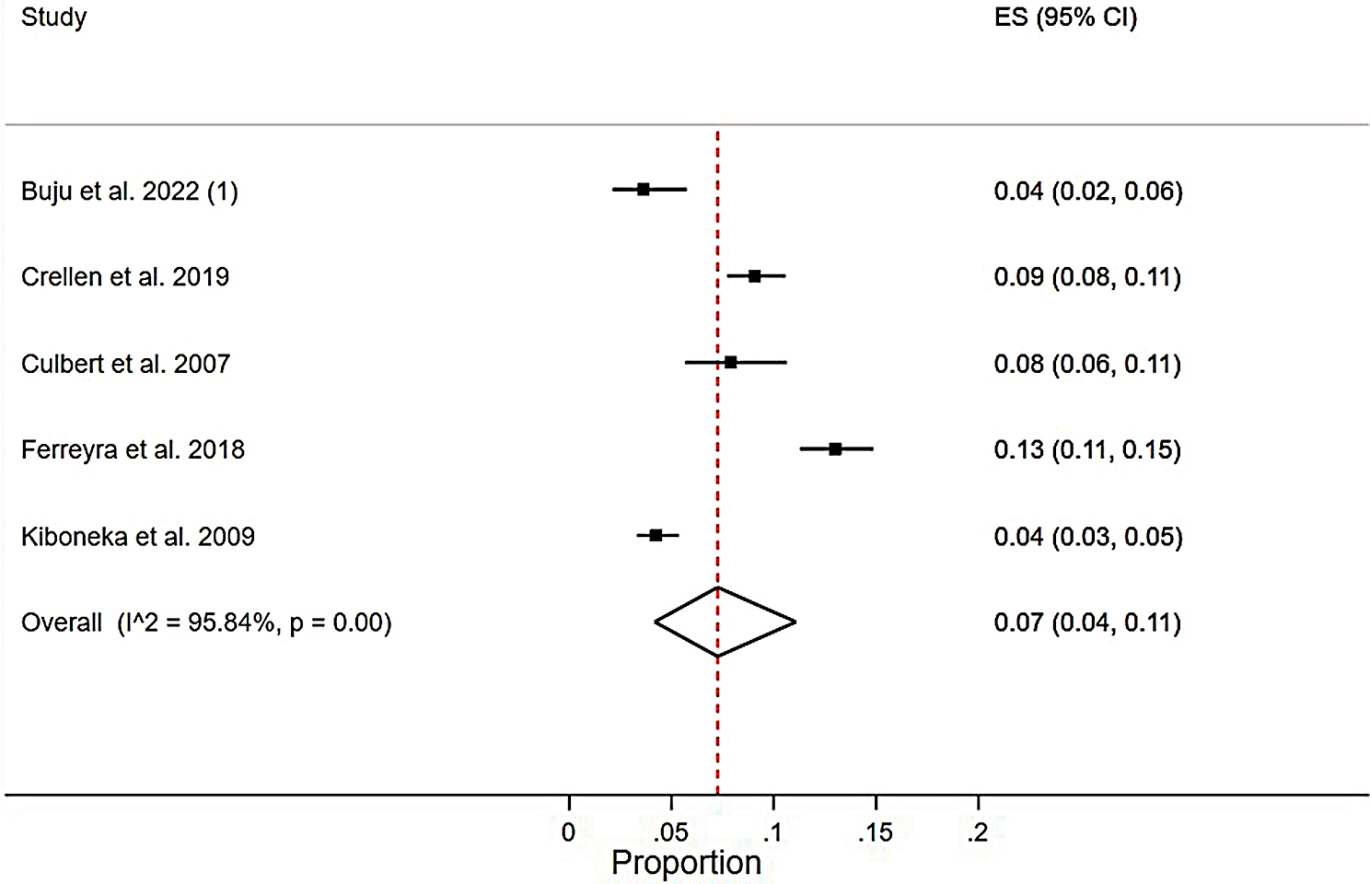
Pooled meta-analysis of mortality rate from active conflict settings among HIV patients who were on ART in SSA from 2002 to 2022

A pooled-analysis of five studies from active conflict settings showed a pooled mortality rate of 0.07 (0.04–0.11), I2 = 95.84%, *p* = 0.00), indicating high clinical heterogeneity (Fig. 8).

## Discussion

This systematic review included 16 articles published from 2002 to 2022. To the best of our knowledge, this systematic review is one of the first comprehensive reviews to assess quantitative studies in seeking to address the substantial gaps in current knowledge regarding the impact of armed conflict on HIV care outcomes in SSA.

Despite the anticipated challenges in accessing ART medication and care schedules in conflict settings[16,32,48], such as ARV medications running out, lack of or poor access to health services, a shortage of medical professionals, and an increased burden of other medical priorities, along with the expected negative impact on HIV care outcomes[16,32,49]; the systematic review found lower rates of non-adherence, loss to follow-up, virologic non-suppression, and mortality compared to those reported from politically stable and well-resourced regions in Sub-Saharan Africa[43,50–54].

Furthermore, the pattern of unfavourable clinical outcomes among those HIV patients in ART reported in the included studies is driven more strongly by patient level covariates than by the evolution of the surrounding long-term conflict, socioeconomic factors[35,39], socioeconomic and clinical factors[33,37,42,46], and clinical variables[34,36,40], and these findings correspond to those of previous research in low-income countries, which also found that unfavourable clinical outcomes were associated with patient age, baseline VL, and the status of treatment before enrollment [53]. This suggests that the cause of poor clinical outcomes was likely not to have been related to armed conflict and associated factors.

The inconsistency of the studies with the perceived outcomes reflects the complex interrelationship between conflict and HIV care. Many factors could have contributed to the disparity in perceived HIV care outcomes and conflict. These include, patient commitment and creativity in obtaining treatment[32,34,38,39,42], as well as education about the benefits of treatment, contribute to positive clinical outcomes. Furthermore, the results of the included studies might have been influenced by information bias, as the impact of conflict-related factors on HIV care was not taken into account. conflict could lead to a range of indirect factors that negatively affect the HIV care outcomes; such as, lack of access to safe drinking water, food insecurity, social unrest, displacement, insecurity, and destruction of livelihoods, psychosocial trauma, and the inability of health systems and other social services[16,39,48,55].

Additionally, the studies themselves had limitations, including retrospective design which rely heavily on patient charts which may have missing data and possible information bias, simplistic study (cause and effect) approach, bias in representativeness as majority of the studies were reported from NGOs funded and managed IDP sites, and a focus on adult populations rather than vulnerable groups like children. These non-governmental organizations (NGOs) are concentrated in small, stable areas. They had a small number of patients in them. The studies were also limited by their small geographic scope. The majority of HIV-infected people, however, live in remote villages.

**Fig. 9:**
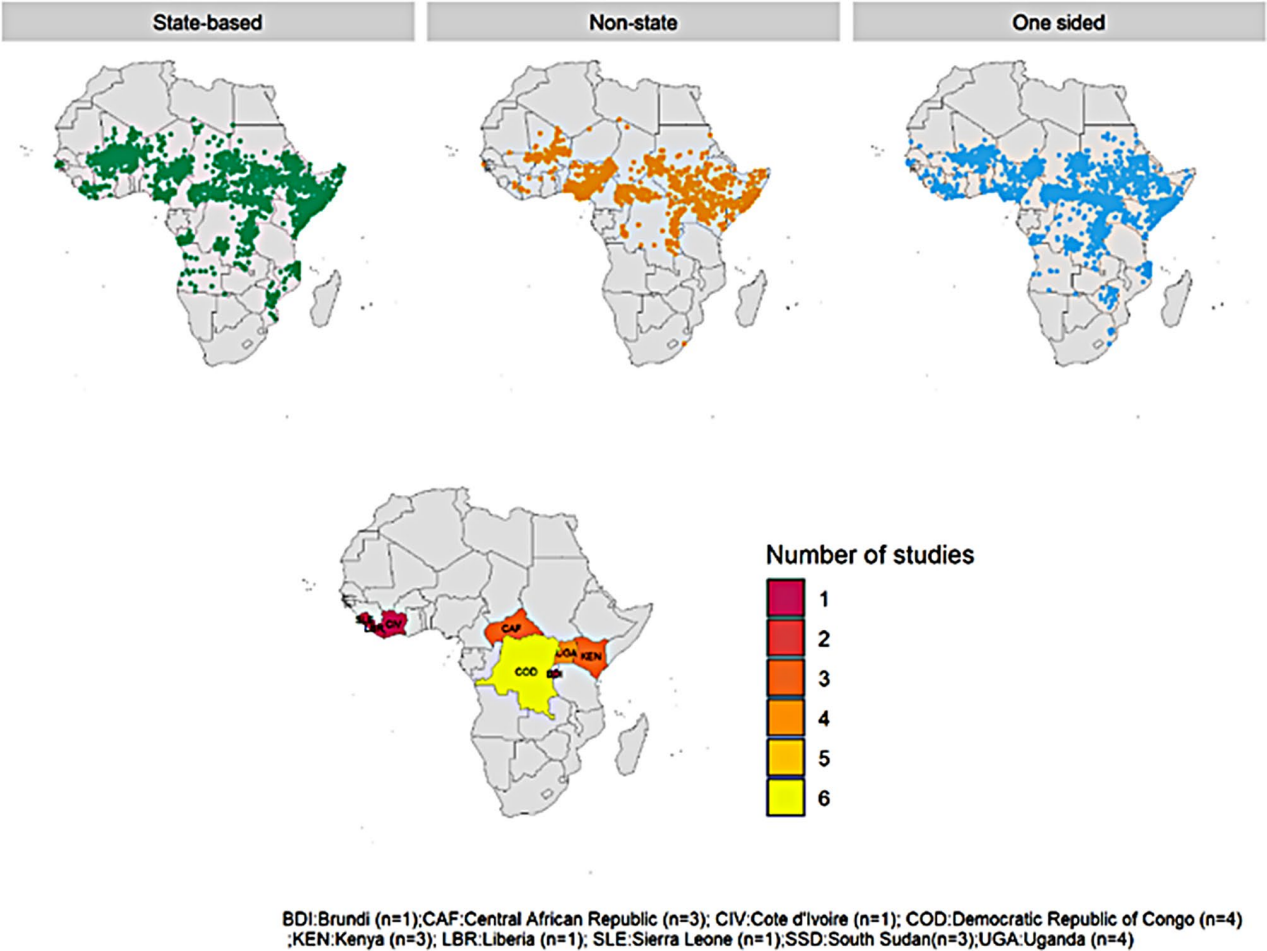
Mapping conflict events, and locations where studies on the impact of armed conflicts on HIV care outcomes were published, SSA between 2002 and 2021. (source: UCDP Version 21)

Therefore, the systematic review highlights the limited research on the relationship between armed conflict and HIV care outcomes in Sub-Saharan Africa. Despite high double burden of armed conflicts and HIV infection in Sub-Saharan Africa, the extensive systematic search revealed that there is limited research on the relationship between armed conflict and HIV care outcomes in Sub-Saharan Africa, with only 16 eligible studies found (Fig. 9). This could be due to the belief that providing ART and conducting research during conflict is too difficult [48]. Furthermore, the existing studies focus on a limited geographic scope and adopt a simplistic cause-and-effect approach, failing to capture the complex and contentious relationship between conflict and HIV care outcomes in SSA. Additionally, the available studies failed to consider the conflict related factors, and indirect factors, post conflict settings. Additionally, pediatrics and mothers were also neglected in the available studies. The available studies fall short of addressing the realities of HIV care in conflict settings, and more research is needed to understand the impacts of armed conflicts on HIV care outcomes, especially in sub-Saharan Africa. Therefore, more research is needed to understand the reciprocal relationship between conflict and HIV care outcomes in Sub-Saharan Africa. Details of the recommended research areas are tabled in Table 2.

**Table 2:** Recommendations for future research

Theme	Recommendation	Stakeholder(s)
Monitoring HIV care	1. Report the approaches and outcomes of HIV care and treatment, as main actors in conflict settings	Health care providers; MOH; donors
Health systems	2. Analyze the existing health system in the context in which services are provided	MOH; health care providers; NGOs
Research	Future research 3. Should consider the severity, scope, onset, and type of conflict.	Researchers, NGOs, MOH
	4. Investigate the indirect mechanisms (malnutrition, comorbidities, and other confounders)	
	5. More emphasis on paediatric HIV/AIDS and PMCTC research	
	6. The need to stimulate new interest in this area of research which covers all conflict affected SSA countries	
	7. Develop valid and reliable data collection system and tools	

## Conclusion

This systematic review filled a gap in study on HIV treatment outcomes in conflict zones in Sub-Saharan Africa. However, the review highlights a lack of research on the relationship between armed conflicts and HIV care outcomes in Sub-Saharan Africa. The available documents lack quality of designs and data sources, and depth and diversity of subjects covered; calling further primary studies on a prioritized future research agenda. Furthermore, there were possible limitations to the review, including there might be exclusion of studies not labeled as conflict-related, selection bias due to language limitations (the database search was limited to English), and potential confounding variables of which we are unaware might be present.

### Electronic supplementary material

Below is the link to the electronic supplementary material.

Supplementary Material 1

Supplementary Material 2

Supplementary Material 3

## Data Availability

All data relevant to the study are included in the article or uploaded as supplementary information. All data relevant to the study are included in the article.

## Declarations

Ethical clearance: Ethical clearance was not needed as this is based on published documents.

### Patient and public involvement

No applicable.

## Abbreviations

ART: Anti-Retroviral Therapy
HIV: Human Immuno-Deficiency Virus
IDP: Internally Displaced People
LTFU: Lost To Follow-Up
MoH: Ministry of Health
NGOs: Non-Governmental Organizations
PRISMA: Preferred Reporting Items for Systematic Reviews and Meta-Analyses (PRISMA)
SSA: Sub-Saharan Africa
