# Supplementary material for: The impact of armed conflicts on HIV treatment outcomes in Sub-Saharan Africa: a systematic review and meta-analysis
Source: Confl Health. 2024 May 17;18:40. doi: 10.1186/s13031-024-00591-8 (PMC11100029; doi:10.1186/s13031-024-00591-8)
Supplement: Supplementary file 1 — Supplementary Material 1 [file 13031_2024_591_MOESM1_ESM.docx]

Supplementary Table 1: Descriptions of quantitative studies about the impact of armed conflicts on HIV treatment outcomes in SSA, 2002-2022

| **S. No** | **Author et al, publication year, Country** | **Study type** | **Population and comparison group** | **Context** | **Year of cohort observation** |
| --- | --- | --- | --- | --- | --- |
| 1 | Ocero, 2009 ^61^, Uganda | Retrospective cohort  (Two years follow-up period) | All HIV-positive patients registered and ever enrolled in to ART  (Participants 402)  Sex (n = 396)  Female 242  Male 154  Age range: was 30 – 44 years | Post-Conflict | 2004 to 2008 |
| 2 | Akilimali et al. 2017 ^55^, DRC | Retrospective cohort  (Every 3 months follow up schedule for … months) | Patients aged >18 years who were in ART programs  Gender (n = 717)  Male 238(33.2)  Female 479(66.8)  Age (mean 39.8± 9.6 years) | Post-conflict | 2004 to 2012 |
| 3 | Buju, R.T et al. 2022 ^53^, DRC | Observational prospective cohort  (Follow up period = 12months) | 468 adults Patients who were aged 18 or older living with HIV and receiving DTG-containing ART  Female 325  Male 143  The average age of the participants was  38.97 years | Conflict affected | 2019 – 2021 |
| 4 | Buju, R.T et al. 2022 ^57^, DRC | Prospective cohort  (Follow up period = 6 months, 12 months) | 305 adults living with HIV and receiving DTG-containing ART  There were 305 records that included viral load results at 6–12 months.  (Patients who were aged 18 or older)  Female = 325  Male = 143  Average age = 38.97 years | Conflict affected | 2019 – 2021 |
| 5 | Buju, R.T et al. 2022 ^56^, DRC | Prospective cohort study  (Follow up period = 1, 3, 6, 9 and 12 months) | 468 adults HIV Patients who were aged 18 or older and receiving DTG-containing ART  (Pregnant women were excluded).  Female = 325  Male = 143  Average age = 38.97 years | Conflict affected | 2019 – 2021 |
| 6 | Crellen et al. 2019 ^66^, CAR | Prospective cohort study  Reports of HIV patient outcomes generally focus on summary statistics after defined intervals (6 or 12 months) and are rarely explored as a detailed time series. | 1631 HIV positive patients including Infants testing positive for HIV after birth  Female = 1147  Male = 484  Median age at first appointment (IQR) 29 years | Conflict setting | 2011 – 2017  18th October 2011 until 31st May 2017  6 months, 60 months |
| 7 | Culbert H. et al. 2007 ^52^, DRC | Retrospective  cohort study  by chart  review  (6 months, 12 months follow up schedule) | 494 patients who had commenced ART.  Female = 326/494 (66%)  Male = 168/494 (44%)  Median age = 37 years | Conflict setting | May 2002-Jan  2006 |
| 8 | Ferreyra et al. 2018 ^67^, CAR | Case study  (Follow up schedule: every 1–3 months) | 1440 patients-initiated ART at MSF facilities | Conflict setting | 2008 – 2016 |
| 9 | Garang PG et al. 2009 ^54^, Uganda | Cross-sectional study | 200 adult patients on ART aged 18 years and above  Female = 135  Male = 65  Average age = 21 to 40 years  (63.5%, n= 127). | Conflict setting | January to  February 2008 |
| 10 | Kiboneka et al. 2009 ^62^, Uganda | Prospective cohort study  (Reporting period: 2 years, 7 months) | Overall (n=1625) adults receiving ART, >14 years-old   - Women =1162 - Men =463 | Conflict setting | June 2005-Jan 2008 |
| 11 | Mann et al. 2013 ^60^, Kenya | Retrospective cohort study | 201 patients enrolled and 189 included in the analysis; aged 18 years or older   - Male = 76 - Female = 125 - Median Age (yrs.) = 42 | Post-conflict (post-election violence) | November 2009 and April 2011 |
| 12 | O'Brien et al. 2010 ^63^, SSA (21 Sub-Saharan  Africa) | Retrospective cohort study by chart review  Reporting period: 6 months, 12 months | Conflict-affected 2572 ≥15 years-old adults with 12-month data | Conflict and post-conflict setting  (Conflict = 13 and  Post-conflict = 11) | 2003 – 2009 |
| 13 | Pyne-Mercier et al. 2011 ^59^, Kenya | Retrospective cohort by chart review  (2 months) | 2534 adult patients aged 18 years or above were included in the study  Female =  Male =  Median age =  During the PEV period, 1,065 people were enrolled and receiving medical attention | Post conflict setting (post-election violence)  - | December  2006-February  2007 |
| 14 | Salami et al et al. 2010 ^64^, South Sudan | Retrospective  cohort study  by chart  review  (9 months) | 102 adult patients who were on ART  Female =  Male =  adults age-cut-off not reported | Conflict setting | July 2009-March 2010 |
| 15 | Ssonko C et al. 2017 ^65^, DRC and South-Sudan | descriptive analysis [cohort] | All patients  DRC = 1053  South Sudan = 186 | Conflict setting | 2010 and 2015 |
| 16 | Yoder et al. 2012 ^58^, Kenya | Retrospective cohort  (Reporting period: 14 months) | 1627 HIV-infected children who were on ART, under the age of 14 14 years  Female =  Male =  Median age = | Post-election crisis | June 2007 – December 2008 |
