# Supplementary material for: The impact of armed conflicts on HIV treatment outcomes in Sub-Saharan Africa: a systematic review and meta-analysis
Source: Confl Health. 2024 May 17;18:40. doi: 10.1186/s13031-024-00591-8 (PMC11100029; doi:10.1186/s13031-024-00591-8)
Supplement: Supplementary file 2 — Supplementary Material 2 [file 13031_2024_591_MOESM2_ESM.docx]

**Supplementary Table 2:** Using appropriate JBI appraisal tools, results of critical appraisal of included studies about the impact of armed conflicts on HIV care outcomes in SSA 2002-2022.

| **Selected Cohort studies** | | | | | | | | | | | | | |
| --- | --- | --- | --- | --- | --- | --- | --- | --- | --- | --- | --- | --- | --- |
| **Study ID** | **Q1** | **Q2** | **Q3** | **Q4** | **Q5** | **Q6** | **Q7** | **Q8** | **Q9** | **Q10** | **Q11** | **Rank**  **(%)** | **Overall appraisal** |
| Salami 2010 | Yes | Yes | Yes | Yes | Yes | Yes | Yes | Yes | Unclear | Unclear | Yes | 81.82 | Included |
| Pyne-Mercier 2011 | Yes | Yes | Yes | Yes | Yes | Yes | Yes | NA | Yes | NA | Yes | 81.82 | Included |
| A.A OOCERO 2009 | No | Yes | Yes | Yes | Yes | Yes | No | Yes | Yes | NA | Yes | 72.73 | Included |
| O'Brien 2010 | Yes | Yes | Yes | Yes | Yes | Yes | Yes | Yes | Yes | Unclear | Yes | 90.91 | Included |
| Culbert 2007 | Yes | Yes | Yes | Yes | Yes | Yes | Yes | Yes | Yes | NA | Yes | 90.91 | Included |
| Crellen 2019 | Yes | Yes | Yes | Yes | Yes | Yes | Yes | NA | Yes | NA | Yes | 90.91 | Included |
| Buju 2022 | Yes | Yes | Yes | Yes | Yes | Yes | Yes | Yes | NA | Yes | Yes | 90.91 | Included |
| Buju 2022 | Yes | Yes | Yes | Yes | Yes | Yes | Yes | Yes | NA | Yes | Yes | 90.91 | Included |
| Buju 2022 | Yes | Yes | Yes | Yes | Yes | Yes | Yes | Yes | NA | Yes | Yes | 90.91 | Included |
| Akilimali 2017 | Yes | Yes | Yes | Yes | Yes | NA | Yes | Yes | Yes | NA | Yes | 90.91 | Included |
| Yoder 2012 | Yes | Yes | Yes | Yes | Yes | Yes | Yes | Yes | NA | NA | Yes | 81.82 | Included |
| Mann 2013 | Yes | Yes | Yes | Yes | Yes | Yes | Yes | Unclear | Yes | NA | Yes | 81.82 | Included |
| Kiboneka 2009 | Yes | Yes | Yes | Yes | Yes | Yes | Yes | Yes | Yes | Yes | Yes | 100.00 | Included |
| Ssonko C 2017 | Yes | Yes | Yes | Yes | Yes | Yes | Yes | Yes | NA | NA | Yes | 81.82 | Included |

| **Case control study** | | | | | | | | | | | | |
| --- | --- | --- | --- | --- | --- | --- | --- | --- | --- | --- | --- | --- |
| **Study ID** | **Q1** | **Q2** | **Q3** | **Q4** | **Q5** | **Q6** | **Q7** | **Q8** | **Q9** | **Q10** | **Rank** | **Overall appraisal** |
| Ferreyra 2018 | Yes | Yes | Yes | Yes | Yes | Unclear | Yes | Yes | Yes | Yes | 90.00 | Included |

| **Cross sectional studies** | | | | | | | | | | |
| --- | --- | --- | --- | --- | --- | --- | --- | --- | --- | --- |
| **Study ID** | **Q1** | **Q2** | **Q3** | **Q4** | **Q5** | **Q6** | **Q7** | **Q8** | **Rank** | **Overall appraisal** |
| Garang 2009 | Yes | Yes | **No** | Yes | Yes | Yes | **No** | Yes | **75.00** | Included |

*Appropriate appraisal for cohort (prospective and retrospective) study, cross sectional study, and case study was used

Y= yes, N= no, NA= not applicable
