# Supplementary material for: The impact of armed conflicts on HIV treatment outcomes in Sub-Saharan Africa: a systematic review and meta-analysis
Source: Confl Health. 2024 May 17;18:40. doi: 10.1186/s13031-024-00591-8 (PMC11100029; doi:10.1186/s13031-024-00591-8)
Supplement: Supplementary file 3 — Supplementary Material 3 [file 13031_2024_591_MOESM3_ESM.docx]

Supplementary Table 2: Main findings of each included document about the impact of armed conflicts on HIV treatment outcomes in SSA, 2002-2022

| **S. No** | **Author, publication year, Country** | **Summary of findings** | | | | |
| --- | --- | --- | --- | --- | --- | --- |
|  |  | **Adherence (retention in care)** | **LTFU, and/or TI** | **CD4 gain** | **Virologic non-suppression, and or/ drug resistance** | **Mortality** |
| 1 | Ocero, 2009 ^61^, Uganda | **Retention rate:** the two-year retention rate was 56.5 %  ***Odds of retention rate:*** The health care setting had a strong correlation with patient retention   - Patients who received ART at the district hospital were two times as likely (OR 0.48 95% CI 0.22, 1.07) to remain in care than those who received it at the health center, and they were five times more likely to do so (OR 0.21 95% CI 0.08, 0.50) than those who received it at the regional hospital | **LTFU:** 175/402 (43.5%) patients were lost to follow-up by the 24th month in care. After three months, the majority of patients were lost to follow-up, and the fewest were after 24 months.  factors associated with LTFU include WHO functional status   - Loss to follow-up was 16 times more likely in the bedridden functional status (OR16.3 95% CI2.0, 132.2) and 3 times more likely in the ambulant patient when compared to those who could work |  |  |  |
| 2 | Akilimali et al. 2017 ^55^, DRC |  | **LTFU:** The median duration of follow-up was 3.99 years (IQR = 2.33 to 5.59). By the end of the study, 86 (12%: 95%CI: 9.614.4) of 717 eligible patients were LTFU.  ***Hazard of LTFU:*** predictors of LTFU   - Patients with a secondary or higher education level (AHR 1.60, 95% CI 1.022.53), - patients living outside of Goma (AHR 1.97, 95% CI 1.023.77), and patients who did not share their HIV status were all more likely to be LTFU |  |  |  |
| 3 | Buju, R.T et al. 2022 ^53^, DRC |  |  |  |  | **Mortality:** With an overall incidence rate of 6.70 deaths per 1000 people per year, the mortality rate for PLHIV on ART at 12 months was 4.9% (95% CI: 3.3-7.3)  ***Hazard of death:*** predictors of mortality   - Older patients had a higher mortality risk when compared to the reference group of 35-year-olds (aHR for the 35-45 age group: 5.14; 95% CI: 1.09-24.18; aHR for the >45 age group: 5.47; 95% CI: 1.13-26.38) - Participants who had never received ART and had a baseline VL of 50 copies/mL (i.e., were suppressed) had a higher mortality risk than those who had (aHR: 5.43; 95% CI: 1.59-18.48)   Participants had a higher mortality risk than the reference group if their baseline VL was high (>1000 copies/mL; aHR: 6.04; 95% CI: 1.78-20.43) |
| 4 | Buju, R.T et al. 2022 ^57^, DRC |  |  |  | **Viral suppression:**   - Nearly three in four patients were virally suppressed at 6 to 12 months and they had a viral load (VL) of <50 copies/ml. after 6 months   ***Odds of non-suppression:*** factors associated with non-suppression   - Adults in the 25-34 age group and those who self-reported as naive patients with a baseline VL of 50 copies/mL and - Abnormal serum creatinine levels had a significantly lower likelihood of not being suppressed (AOR = 0.33, 95% CI 0.12-0.93) - Advanced disease stage; being in stage III or IV of the disease (AOR = 1.86, 95% CI 1.01-3.43) - High viral load; those who had a high baseline HIV viremia of over 1000 copies/mL (AOR = 3.41, 95% CI 1.64–7.08)   Ethnic; the ethnically Sudanese population (AOR = 4.19, 95% CI 1.43–12.68) compared to their reference groups |  |
| 5 | Buju, R.T et al. 2022 ^56^, DRC |  | **Rate of LTFU:**   - Over one-fourth (28, 8%; 95% CI: 24.9-33.1) of the cohorts were LTFU during the study period - After 1, 3, 6, 9 and 12 months, patients were lost to follow-up in proportions of 12.0%, 21.4%, 26.5%, 28.6% and 28.8%.   ***Odds of LTFU:***   - Patients who were less experienced on ART at enrollment (48% vs. 61%; p < 0.001)   Being Sudanese national had a higher risk of being LTFU compared the reference group |  |  |  |
| 6 | Crellen et al. 2019 ^66^, CAR |  | **LTFU:** 183/1631 (11.2%) patients were loss to follow up **(11.2% over 5 years)** | the recovery of CD4-cell counts |  | Overall, 1631 patients were enrolled and 1628 were included in the analysis giving 145 deaths.  **Mortality:**   - The baseline monthly risk of mortality declined the longer the patient remained in the cohort - The risk of mortality for individual patients was highest in the second month after entering the cohort (0.0010 (95% CrI 4.7 × 10− 4, 0.0023)), and declined seven-fold over the first 12 months (1.5 × 10− 4 (95% CrI 5.8 × 10− 5, 3.9 × 10− 4))   ***Odds of mortality:*** factors associated with a higher monthly risk of mortality   - Sex: Male Vs female; (OR) 1.7 (95% CrI 1.2, 2.3) - Age: higher age Vs lower age; OR 1.01 (95% CrI 1.00, 1.03) for each unit (year) increase. - Higher WHO disease stage: stage 1 OIs, stage 2 OIs had an OR of 2.5 (95% CrI 2.0, 3.2), stage 3 OIs had an OR of 6.4 (95% CrI 4.1, 10) and stage 4 OIs had an OR of 16 (95% CrI 8.2, 34) |
| 7 | Culbert H. et al. 2007 ^52^, DRC | Number of patients initiating ART = 494  **Adherence:** >95% pills taken as prescribed as of last clinic visit, measured by pill counts=99% | **LTFU:** 12-month loss to follow-up [95% CI] = 5.4% [3.2–7.5] | **Immunologic gain:** 6-month median CD4 gain, cells/ml [IQR] = 163 [82–232] |  | **Mortality:** 12-month mortality [95% CI] = 7.9% [3.6–12.1] |
| 8 | Ferreyra et al. 2018 ^67^, CAR | A total of 1440 patients-initiated ART.  **Retention in care:** 782/1440 (54%) patients started on ART were still under active follow up, | **LTFU** 354/1440 (25%) patients had been lost to follow up |  | **Viral load monitoring**: 390 samples were sent and 212 (54%) results were available. 139 (66%) patients were virologically suppressed and 71 (33%) had a viral load higher than 1000 copies/ml. | **Mortality:** 182/1440 (13%) had died. |
| 9 | Garang PG et al. 2009 ^54^, Uganda | **Adherence rate:** The majority of the participants (37.5%) had used ART for longer than 24 months. The mean 4-day self-reported adherence recall was 99.5% (≥95% doses taken as prescribed= 99.5%)  ***Odds of non-adherence (<95% adherence):*** Being on a first-line ART regimen and feeling that the staff at the health center were critical were both independently associated with nonadherence   - First line vs. second line treatment [OR=22.22, 95% CI 1.48-333.33, p=0.03] - Staff were condemning, yes vs. no   (OR=22.22, 95% CI 1.5-333.33, p=0.02) |  |  |  |  |
| 10 | Kiboneka et al. 2009 ^62^, Uganda | **Adherence:** Of the 1521 patients with adherence data, 1403 (92.2%) had adherence of 95% or higher and 118 (7.8%) had less than 95% adherence (≥95% doses taken as prescribed=92.2%) |  | **CD4 gain:** Median CD4 Change (IQR)= 0 (0-0)  Baseline CD4 count, per 100 cell increase (HR=0.14, 95% CI 0.06-0.34, p<0.001) |  | **Mortality:** Of the 1625 patients who participated in the study, 69 (4.2%) died during follow-up. Mortality incidence=3.48 (95% CI 2.66-4.31) per 100 person-years, log rank p-value<0.01  ***Hazard of death:*** Lower mortality was associated with:   - Female sex (HR=0.7 95% CI 0.55-0.91, p=0.02) - Higher baseline CD4count (HR per 100 cells increase 0.14, 0.06 to 0.34, P<0.0001) - 95% adherence (HR 0.14, 0.10 to 0.21, P<0.0001) |
| 11 | Mann et al. 2013 ^60^, Kenya |  |  | **CD4 Count:**   - Between pre-crisis and enrolment, the mean CD4 values increased by 44% and 4% in the interruption group and 25% and 4% in the control group, respectively (P = ns) - In the Interruption group, the mean enrolment CD4 values were 384 and 21%, while in the Control group, they were 421 and 24% (P = 0.18 and 0.04, respectively) | The median time on treatment was 4.3 years and median time after conflict was 2.2 years. One hundred thirteen patients received ongoing treatment, and 88 patients had TIs associated with conflict.   - Patients with TIs were more likely to have detectable VL, VL.5,000, and VL.10,000 (< 400, >400 and < 10,000, and >10,000) compared with those without (AOR, 2.4, adjusted for precrisis CD4; 95% CI: 1.1 to 5.4; P = 0.03)   **Drug Resistance:**  Of 32 patients with detectable VL (20 Interruption and 12 Control), genotypes were available for 17 of the 20 and 11 of the 12 patients  Sixty-five percent in the Interruption group and 64% in the Control group had intermediate or high-predicted resistance to first-line ARVs |  |
| 12 | O'Brien et al. 2010 ^63^, SSA (21 Sub-Saharan  Africa) | Antiretroviral therapy was started in 22 programs with 4555 patients. 2572 patients were with 12-month data. With ART, the median follow-up period was 11.8 months (IQR 3.9-22.7). | **LTFU:** -Proportion of lost to follow-up at 12 months was 466/2572 (11%), 95% CI 0.09-0.12 | **CD4 gain:** Median 6-month CD4 gain: 129 cells/mm3 |  | **Mortality:** 9% Mortality of those on ART, 12 months  (The median 12-month survival for the 2572 (61%) adults with 12-month data was 0.89 (95% CI 0.88-0.91)) |
| 13 | Pyne-Mercier et al. 2011 ^59^, Kenya |  | **TI:** Proportion of patients experiencing treatment Interrupting (visiting Pharmacy ≥48 hours after ARTs completed)   - 16.1% in PEV group - 10.2% in comparison group   ***Odds of TI*:** Odds of TI during PEV increased by 71% 95%CI 34 to 118]   - During postelection violence, odds of TI increased for men (OR=1.37, 95%CI 1.07 to 1.76, p=0.01) and clients travelling ≥ 3 hours to clinic (OR=1.86, 95% CI 1.28 to 2.71, p=0.001) |  |  |  |
| 14 | Salami et al et al. 2010 ^64^, South Sudan | **Adherence:** Of the 102 patients who were on ART for at least 6 months   - 88% reported adherence levels of >95% (had missed less than 3 doses within last month) - Adherence was higher in females (92%) compared to males (80%) |  |  |  |  |
| 15 | Ssonko C et al. 2017 ^65^, DRC and South-Sudan | In Leer (South Sudan), patients fled to the swamps only with their close properties and in many cases, ART was interrupted. All ART clinical files were lost. |  |  | **Viral Load**  At the end of 2015 in DRC, a total of 413 samples were tested in a cohort of 988 patients (42%). In 2015, 78% and 66% of the samples in Baraka and Kimbi respectively were below the threshold of 1000 copies HIV RNA VL/ml. |  |
| 16 | Yoder et al. 2012 ^58^, Kenya | **Medication Adherence**  Adherence: 91.8% (1’494/1627) had perfect adherence 4 months post-election and 91% (1481/1627) had perfect adherence 5-10 months post-election | **LTFU**  LTFU: 3% (49/1627) initial loss to follow up, 2.6% (42/1627) after 12 months |  |  |  |
